# Mycotoxin Determination in Animal Feed: An LC-FLD Method for Simultaneous Quantification of Aflatoxins, Ochratoxins and Zearelanone in This Matrix

**DOI:** 10.3390/toxins12060374

**Published:** 2020-06-05

**Authors:** Borja Muñoz-Solano, Elena González-Peñas

**Affiliations:** Department of Pharmaceutical Technology and Chemistry, School of Pharmacy and Nutrition, Universidad de Navarra, 31008 Pamplona, Spain; bmunoz.1@alumni.unav.es

**Keywords:** feed, mycotoxins, liquid chromatography, fluorescence, multidetection

## Abstract

Mycotoxins are toxic compounds for humans and animals that are produced by fungi. Mycotoxin contamination in feed is a global safety concern and effective control of these compounds in this matrix is needed. This study proposes a simple, cost-effective analytical method based on liquid chromatography coupled with a fluorescence detector, which is suitable for the routine monitoring of some of the most important mycotoxins in feed: aflatoxins (G2, G1, B2, and B1), zearalenone, and ochratoxins A and B. Mycotoxin extraction, chromatographic separation and quantification are carried out simultaneously for all mycotoxins. The extraction procedure is performed using acetonitrile, water and orthophosphoric acid (80:19:1). Purification of the extract is carried out using an OASIS PRIME HLB solid-phase extraction cartridge followed by a dispersive liquid–liquid microextraction procedure. Aflatoxins G1 and B1 are derivatized post-column (photochemical reactor at 254 nm) to increase their signal. The method has been validated in feed for pigs, cows, sheep, and poultry with very satisfactory results. The detection limits are 2 μg/kg for aflatoxins B1 and G1, 0.64 μg/kg for aflatoxins B2 and G2, 42 μg/kg for zearalenone, and 5 μg/kg for ochratoxins A and B. These values are low enough to allow for monitoring of these mycotoxins in feed. Global recovery values were between 73.6% and 88.0% for all toxins with a relative standard deviation (RSD) % < 7%. This methodology will facilitate laboratory control and analysis of mycotoxins in feed.

## 1. Introduction

The value of animals for food production is approximately €130 billion in the European Union (EU) [1]; animal diseases and health problems that reduce production of animal derived products have a major impact on global economics and trade. One more very important reason for maintaining animal health is also to protect consumer health. Some contaminants can enter the human food chain through food-producing animals, and if feed is contaminated and not monitored, it may have an impact on food safety [2]. Animal health is therefore a major challenge worldwide and must be protected, especially through adequate nutrition [3].

The EU define feed as “any substance or product, including additives, whether processed, partially processed or unprocessed, intended to be used for oral feeding to animals” [2]. Each year, animals for food production in the EU required approximately 450 million metric tons of feed [1]; and the growth in animal production will increase the demand for this product.

Animal feed is produced using a highly complex mix of water, lipids, fatty acids, proteins, amino acids, carbohydrates, minerals, vitamins, and other components, all of which are required to ensure the health and productivity of farm animals. Feed composition varies depending on the age and species of animal for which it is intended. The different components are obtained from diverse agriculturally produced raw materials, especially cereals and grains. Feed is a main parameter in every animal production system [4] and is the main source of expenditure in farms [5].

Agricultural raw materials can be contaminated by fungi during the growing process, prior to harvest, or during storage in inadequate conditions of humidity and/or temperature [6]. Fungi produce mycotoxins as secondary metabolites. *Aspergillus*, *Fusarium*, *Penicillium,* and *Claviceps* are the main mycotoxin producers [7]. Deoxynivalenol and zearalenone (ZEA) have been reported at rates of 60% and 80%, respectively, in food grains from 2010 to 2015 in the EU [8]. It should be noted that mycotoxins may be present in raw materials or feed, even if molds are no longer present, due to their resistance to several environmental factors [9]. Moreover, new techniques used in intensive agriculture, together with climate change and market globalization, will lead to unpredictable changes in the occurrence of molds in grains and toxin distribution around the world [6,9]. It should also be noted that, due to regulation of the presence of mycotoxins in human food, in some cases, compounds that are not suitable for human consumption may be used in feed preparation [4].

Mycotoxins are toxic to animals and humans and their presence in feed is a global safety concern [7,10]. Aflatoxins (AFs), ochratoxin A (OTA), and ZEA are considered to be of particular concern in feed [11]. Mycotoxins have several effects on animal health, such as structural and functional damage to the liver, nephrotoxicity, hepatotoxicity, and immunotoxicity, in addition to poor weight gain in animals and decreased egg or milk production [6,11]. Furthermore, some of these toxins, such as ZEA, have estrogenic activity and cause reproductive problems in animals, including hyperestrogenism, sterility, and abortions [10]. AFs, especially aflatoxin B1 (AFB1), have been classified as human carcinogens (group I) by the International Agency for Research on Cancer [12], and OTA, fumonisins and sterygmatocistin have been classified as possible human carcinogens (Group 2B) [13,14]. Moreover, exposure to several mycotoxins may lead to synergistic effects [6]. For instance, simultaneous contamination of feed with AFs and ZEA reduces egg quality and production and also decreases feed intake in hens [15].

Despite efforts to reduce the presence of mycotoxins in feed, a review published in 2013 on the occurrence of mycotoxins in feed and feed raw materials worldwide over an 8-year period, including more than 17,000 samples, found mycotoxins in 72% of the analyzed samples, and two or more mycotoxins were found in 38% of the samples [6]. Other studies have also reported mycotoxins in a high percentage of analyzed feed samples and have confirmed co-occurrence [9,16]. Although low levels were found, some samples presented levels above maximum permitted limits [16].

Finally, an economic impact of mycotoxins in animal production comes from animal diseases, losses in animal productivity, rejection of feedstuffs, and from costs derived from prevention, research, etc. [17].

For all of the above reasons, and because of the problem that mycotoxin contamination of feed represents, control and monitoring of the presence of mycotoxins in animal feedstuffs is essential in order to maintain low levels and prevent highly contaminated feed from reaching the animal food chain. To this end, the EU has established maximum permitted levels of some mycotoxins in feed. These levels are as follows: ZEA, 100–500 µg/kg for complementary and complete feeding-stuffs and 2–3 mg/kg for feed material; OTA, 50–100 µg/kg for complementary and complete feeding-stuffs and 0.25 mg/kg for feed material; and 5–20 µg/kg of AFB1 depending on the feed material [18,19]. These levels should be routinely monitored using validated, reliable, robust, fast, and inexpensive analytical methods [20].

Several authors have described different methodologies for achieving a quantitative determination of mycotoxins in different raw materials and feed matrices. Liquid chromatography (LC) is the most commonly used technique [21], especially when coupled with a mass spectrometry detector (MS) [22,23] or a fluorescence detector (FLD) [5,24,25,26].

The trend in recent years is to use an MS detector, especially for determining multiple mycotoxins. This is due to the different advantages of MS, such as the fact that it is a universal detector, with very highly selective and sensitive detection [27]. However, one of its main drawbacks is the high cost of analysis and considerable training required by analysts. For these reasons, many small companies and control laboratories that perform analytical screening of feed and raw materials have difficulty accessing this technology, thus making it advisable to use simpler alternatives such as FLD, coupled to simplified sample preparation procedures. Moreover, these alternatives should achieve the multi-detection of several mycotoxins, even if they belong to different chemical families.

**Figure 1:**
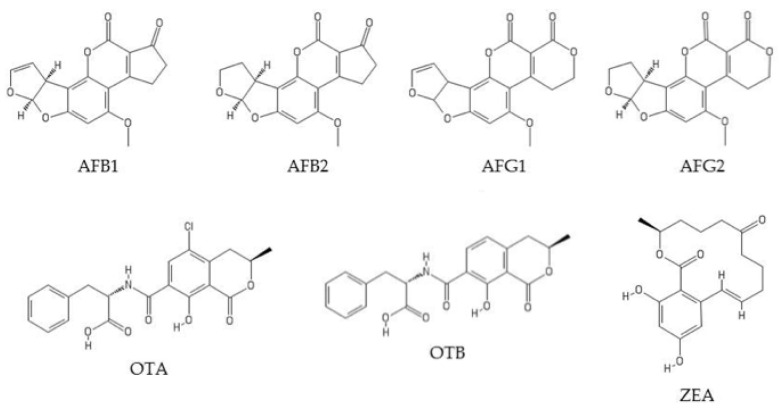
Chemical structures of aflatoxin B1 (AFB1), aflatoxin B2 (AFB2), aflatoxin G1 (AFG1), aflatoxin G2 (AFG2), ochratoxin A (OTA), ochratoxin B (OTB), and zearalenone (ZEA). Adapted from [28].

In this study, we present a validated analytical methodology using LC-FLD for the simultaneous quantification of AFB1, aflatoxin B2 (AFB2), aflatoxin G1 (AFG1), aflatoxin G2 (AFG2), OTA, ochratoxin B (OTB), and ZEA in animal feed. The structures of these mycotoxins are shown in Figure 1.

The developed method shows as innovative aspects the use of LC-FLD technique, widespread in most control laboratories, and the simultaneous analysis of seven mycotoxins with different chemical characteristics. Also, this procedure has proven to be suitable for use in four different type of feeds: for cows, pigs, sheep, and poultry. It has a quick and easy procedure for sample processing, adequate limits of detection and quantification, reliable results, and cost-effective analysis.

## 2. Results

### 2.1. Sample Treatment

**Figure 2:**
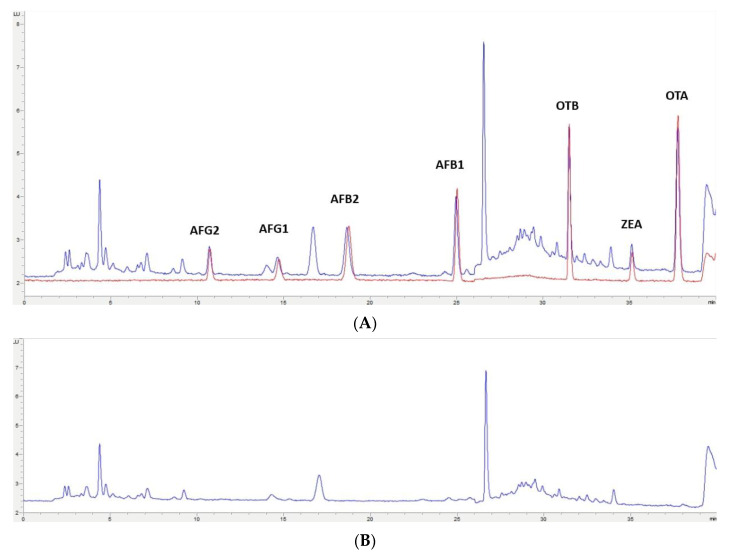
(**A**)**:** Superposition of a chromatogram of a feed sample for poultry fortified at 2× limit of quantification (LOQ) level and one from a calibrator at the same concentration level. (**B**): Chromatogram extract of a blank feed sample for poultry.

**Figure 3:**
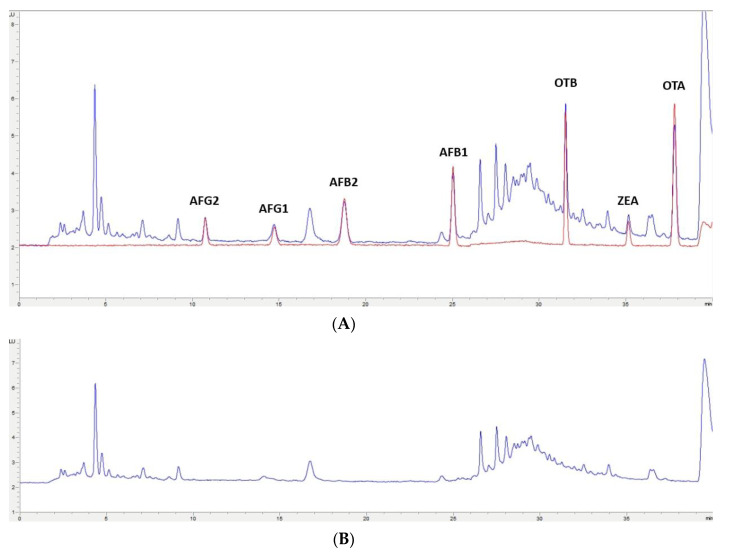
(**A**)**:** Superposition of a chromatogram of a feed sample for cow fortified at 2× LOQ level and one from a calibrator at the same concentration level. (**B**): Chromatogram extract of a blank feed sample for cow.

**Figure 4:**
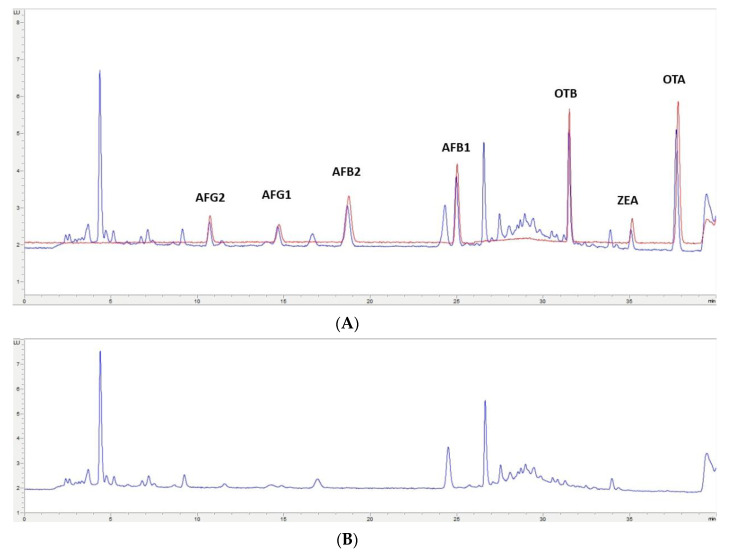
(**A**)**:** Superposition of a chromatogram of a feed sample for pig fortified at 2× LOQ level and one from a calibrator at the same concentration level. (**B**): Chromatogram extract of a blank feed sample for pig.

**Figure 5:**
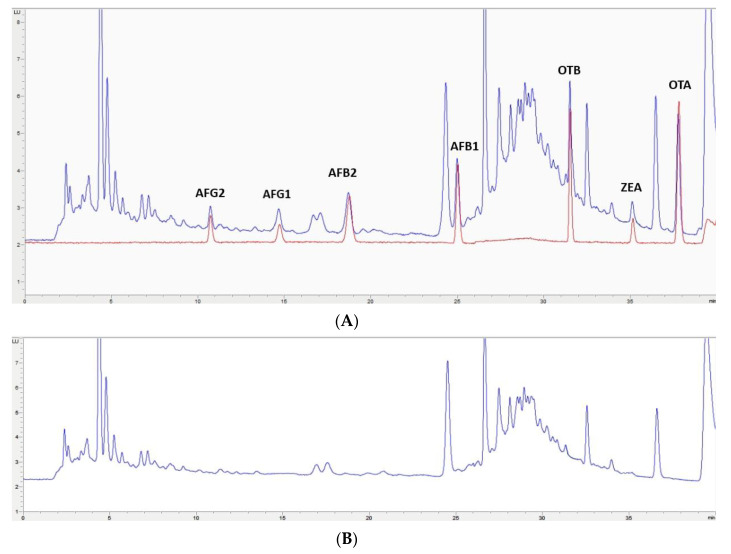
(**A**)**:** Superposition of a chromatogram of a feed sample for sheep fortified at 2× LOQ level and one from a calibrator at the same concentration level. (**B**): Chromatogram extract of a blank feed sample for sheep.

In this study, the extraction of mycotoxins from feed samples was first attempted using a Quick, Easy, Cheap, Effective, Rugged and Safe (QuEChERS) methodology. The first approach involved an extraction using water and a solution of acetonitrile (ACN)/formic acid (99/1) or orthophosphoric acid. NaCl and MgSO_4_ were then added and after centrifugation, a supernatant containing the extracted mycotoxins could be separated. However, the extract was not clean enough for chromatographic analysis; also, no differences were observed when using formic or orthophosphoric acid. Then, to improve the cleaning-up of the extract, the supernatant was treated with MgSO_4_ and C_18_ particles in different proportions, but this process was not successful. Moreover, primary/secondary amine (PSA), alumina, and active carbon were assayed, but very low recovery values (lower than 60% for OTA, OTB, AFG1, and AFG2) were obtained. Finally, QuEChERS was discarded and an OASIS PRIME HLB solid-phase extraction (SPE) (Waters, Milford, MA, USA) cartridge in pass-through mode was used. The eluate obtained went through a second clean-up process based on dispersive liquid–liquid microextraction (DLLME) with chloroform and water. Based on the procedure used by Zhou et al. [29], to achieve multi-detection of mycotoxins in cereals and bean foodstuffs, a new DLLME procedure was developed because some modifications were required to adapt it to feed samples. This procedure of sample treatment gave very clean extracts for each one of the assayed matrixes; also, similar chromatograms have been obtained from each type of non-fortified feed (Figure 2B, Figure 3B, Figure 4B, Figure 5B). These results indicate that this methodology would have a wide applicability for mycotoxin analysis in feed.

### 2.2. Chromatographic Conditions

The selected chromatographic conditions produced good separation between the mycotoxins themselves and any other compounds in the extract, as shown in the two chromatograms in Figure 2A, Figure 3A, Figure 4A, Figure 5A. One of them corresponds to a chromatogram obtained from a feed sample that was fortified at 2× limit of quantification (LOQ). The superposed chromatogram corresponds to one obtained after analysis of a calibrator at the same concentration. Figure 2B, Figure 3B, Figure 4B, Figure 5B shows a chromatogram obtained from a non-fortified feed sample. Retention times (min) obtained for each mycotoxin at three levels of concentration (LOQ, 2.5× LOQ, and 5× LOQ) in selectivity studies had a relative standard deviation (RSD, %) < 0.28% and the retention times of mycotoxins in the fortified samples corresponded to those in the calibrators with a relative error (RE) < 0.87% (Table S1).

### 2.3. Method Validation

All the validation parameters studied met the previously established validation criteria. The calibration curves started with the LOQ level, for which acceptable precision and trueness results were obtained. All the curves demonstrated linearity and met the fixed criteria: back-calculated concentrations differed less than 15% from the respective nominal values; slopes also met the linearity criteria (Table 1 and Table S2).

Furthermore, calibrator precision and trueness were less than 8% and 14%, respectively, under within-day and between-day conditions (Table 2). Instrumental precision was also adequate, as after 10 injections of a calibrator at 2.5× LOQ, the RSD (%) of the peak areas for each mycotoxin was less than 2% in all cases, whereas the RSD (%) for retention time was less than 1% in all the mycotoxins.

Recovery was obtained for each mycotoxin at three concentration levels under within-day (*n* = 3) and between-day (*n* = 9) conditions and in the four types of feed studied (Table 3 and Table 4 and Tables S3–S6). 

The overall recovery values obtained for each mycotoxin ranged from 73.6% for AFG1 in poultry feed to 88.0% for AFB2 in feed for pigs. In all cases, RSD (%) values were less than 7% (Table 5).

### 2.4. Application

This methodology was applied to mycotoxin analysis of 10 feed samples for the species studied (three for poultry, two for ovine, two for bovine and three for pig). Only ZEA was detected: in all the samples for poultry (112–320 μg/kg) and in one sample for pigs (178 μg/kg) (data corrected for recovery).

## 3. Discussion

The study of contamination of animal feed by mycotoxins is a topic of general interest from two perspectives. It is important at the clinical level due to the adverse effects triggered in animals when mycotoxins enter the animal food chain. Mycotoxins are also a problem from an economic point of view because large quantities of feed and raw materials have to be eliminated when levels of certain legislated mycotoxins are exceeded. This major economic loss affects producers and consumers. Therefore, many authors have worked over the years to develop increasingly more sensitive, precise and robust analytical methods in a number of raw materials, with a strong trend in recent years toward multi-detection of mycotoxins. Multi-detection is a highly desirable characteristic for an analytical method designed to quantify mycotoxins. The presence of different mycotoxins in one raw material is highly likely because a raw material can be infected by several fungi, one fungus can produce more than one mycotoxin and, finally, feed is a complex mix of raw materials. The presence of multiple mycotoxins in feed is therefore common [11,16]. In this scenario, mycotoxin multi-detection provides a great deal of information in one analytical run, reduces the time to obtain results, involves major cost savings, and makes it possible to make much quicker decisions.

The most commonly used methodologies for detecting and quantifying mycotoxins in feed are based on LC with multiple detection systems [11]. One of the most powerful tools is LC coupled with MS in tandem (LC-MS/MS) because of the universality and sensitivity of this detector, which allows for multi-detection of a large number of mycotoxins with different physical and chemical characteristics. It also usually provides the opportunity to obtain important information, such as structural determination in some cases. However, it also has some drawbacks. Detection by MS is strongly conditioned by the matrix components, which can compromise quantification. In addition, this detector is very expensive in terms of the purchase price, personnel training, and maintenance, thus making it inaccessible for many companies and laboratories directly involved in the daily control of mycotoxins in feed. These laboratories need more accessible analytical methodologies to make monitoring simpler and to allow finished feed batches that have been adequately tested for the presence of mycotoxins to reach the market.

The FLD is less expensive than MS and is specific and sensitive, although Zhao et al. indicated that, in different reported methodologies, FLD detector sensitivity is usually not sufficient for AFs determination in feed due to the low levels encountered [30]. The FLD detector is easier and less expensive to use than MS. When this detector is employed, some mycotoxins need derivatization in order to improve the response [31]. This is the case of AFB1 and AFG1 [5] and of fumonisin B1 (FB1) and fumonisin B2 (FB2) [32,33,34]. The FLD has been used in the determination of mycotoxins in several raw materials, such as cereals [35,36,37], even in detection of multiple mycotoxins [38].

For these reasons, we have developed an industry-oriented LC-FLD method that achieves multi-detection of seven mycotoxins in feed chosen for their presence and interest in this matrix.

A search of the literature in PubMed, including the search terms “Mycotoxin AND feed AND fluorescence,” limited to the last 10 years, revealed a total of 20 studies on the analysis of mycotoxins in feed (no raw materials) using LC-FLD [4,5,9,16,17,30,32,33,34,39,40,41,42,43,44,45,46,47,48,49]. AFs are the most widely studied mycotoxins [4,5,9,16,17,30,40,41,42,43], particularly AFB1, as a single mycotoxin [44,47,48,49]. Other mycotoxins studied are OTA [4,9,17,44,45,46], ZEA [17,33,42,44,47], FB1 and FB2 [32,33,34], and T-2 toxin [33]. Only one study achieves multi-mycotoxin quantification (including mycotoxins from different chemical groups) of AFB1, AFB2, AFG1, AFG2, OTA, and ZEA using immunoaffinity columns (IAC) [39]. However, these authors described validation of the method using egg and not chicken feed. The interest of this method compared to other published methods is that the authors achieved simultaneous detection of six mycotoxins. The authors also took advantage of new developments and used one IAC column that retained several toxins instead of using different IACs for each group of mycotoxins, which is common procedure in the articles cited.

Animal feed is a complex mix of different compounds depending on the species for which it is prepared. Sample treatment is needed to remove most of these compounds. In the developed method described in this document, and in most of the published methods, extraction of the analytes from the samples was performed using ACN and water, but in this case, water was acidified with orthophosphoric acid to favor extraction of the mycotoxins to the organic solution, taking advantage of the fact that some of them are weak acids and an acidic medium will favor their pass to the organic solvent.

The clean-up procedure was carried out in two stages. The first stage was based on SPE, thus avoiding the use of IAC. This aspect is one of the advantages of the proposed method. IAC columns are used for sample purification in nearly 90% of the methods developed for mycotoxins in feed in the last 10 years because they provide clean sample extracts and allow the analytes of interest to be concentrated to reduce quantification limits. But these columns have some disadvantages. They are expensive and are usually prepared for a single use [5], which greatly increases the processing price of each sample. Some authors have also reported low recovery values and matrix interferences when no previous clean-up steps were carried out before IAC; this is due to the complexity of the feed matrix [33] and IAC loading capacity [32].

New approaches have been tried in order to avoid the use of IAC. Zhao et al. developed an ionic-liquid-based DLLM that was combined with magnetic solid phase extraction based on 1-octyl-3-methylimidazolium hexafluorophosphate and pelargonic acid coated Fe_3_O_4_ magnetic nanoparticles for AFs extraction from feed. The authors obtained recoveries of greater than 90% and no significant differences between data obtained using this procedure and the IAC clean-up were observed [30]. Arroyo-Manzanares et al. developed a very easy extraction method of AFs based on SPE using ACN while achieving good recovery values (>80%) and the authors described good cleanliness of the final extract [5]. Khayoon et al. used Isoluted Multimode^®^ columns that combined different retention mechanisms (C_18_, strong cation and anion exchange) in order to purify the feed extracts prior to analyze AFs by LC-FLD, and authors concluded that these columns can be an alternative to the more expensive IAC to clean-up extracts from matrix components. Recoveries in mixed feed were greater than 70% for AFB1, AFB2, AFG1, and AFG2 [40].

Other authors [33] used a much more complex extraction procedure, in which the extract was defatted using petroleum ether, then passed through a Florisil column and, finally, through the corresponding IAC. This allowed the authors to obtain recovery values of between 72% and 95% for T-2 toxin, FB1, and ZEA.

The second part of the proposed extraction procedure was based on DLLME with chloroform that achieved a high matrix component cleaning before analysis. The obtained recovery values are good enough for mycotoxin analysis in feedstuffs with adequate RSD values. Moreover, validation demonstrated that the detection limits obtained for all mycotoxins were below the maximum limits proposed by the EU for mycotoxins studied in animal feed [18,19].

The proposed methodology will aid producers who outsource mycotoxin analysis of their feeds, as well as external laboratories, in effective mycotoxin monitoring in feed because of its quick and easy procedure for sample processing, adequate limits of detection and quantification, reliable results, and cost-effective analysis.

## 4. Conclusions

The analytical procedure presented here has been fully validated for the quantification of seven of the most important mycotoxins and those with the greatest impact on the feed production industry: AFG2, AFG1, AFB2, AFB1, OTB, OTA, and ZEA. It is a suitable method for animal feed for sheep, cows, pigs, and poultry. All the validation parameters meet the requirements proposed by the guidelines consulted, as well as our own goal of providing a cost-effective and reliable method. The limits of detection and quantification reported make it possible to detect the maximum legislated values for each mycotoxin in each of the matrices studied. The cleanliness of the matrix components led to good recovery values without the use of IAC. These features and the simplicity of the sample-processing procedure make it possible to analyze a large number of samples in a relatively short period of time and, therefore, this methodology will facilitate laboratory control and analysis of mycotoxins in feed.

## 5. Materials and Methods

### 5.1. Chemical and Reagents

LC-gradient grade acetonitrile (ACN) and methanol (MeOH) were purchased from Honeywell Riedel-de Haën (Muskegon, MI, USA). LC-gradient grade chloroform and water were obtained from Fisher Scientific (Bishop Meadow, UK). Pro-analysis grade orthophosphoric acid was obtained from Panreac (Barcelona, Spain). OASIS PRIME HLB SPE cartridges were obtained from Waters (Milford, MA, USA).

### 5.2. Mycotoxin Standards

AFB1 (PubChem CID: 186907), AFB2 (PubChem CID: 2724360), AFG1 (PubChem CID: 14421), AFG2 (PubChem CID: 2724362), OTA (PubChem CID: 442530), OTB (PubChem CID: 20966), and ZEA (PubChem CID: 5281576) were obtained from Sigma–Aldrich (Merck KGaA, Darmstadt, Germany). All mycotoxins had a purity of 98% or greater. They were obtained in solution in ACN, and only OTA was purchased in powder form. The concentrations of the mycotoxin solutions were as follows: AFG2 and AFB2, 0.5 μg/mL; AFG1 and AFB1, 2 μg/mL; OTB, 10 μg/mL; and ZEA, 100 μg/mL. OTA was dissolved in MeOH at a concentration of 1 mg/mL. Ultraviolet spectrophotometry at 333 nm (UVIKON 922, Kontron Instruments SA, Spain) was used to determine the final concentration of the OTA solution. All solutions were stored at −20 °C until use.

### 5.3. Safety Precautions

Because of their toxicity, mycotoxins were always handled in solution. OTA powder was not weighed and was dissolved in MeOH in the vial in which it was packed. Personal protection measures (gloves and face mask) were used in the laboratory when mycotoxin solutions were handled; for instance, when samples were spiked or in the preparation of calibrators. Low-light conditions were also used to prevent photodegradation of AFs.

### 5.4. Animal Feed Samples

Due to the numerous possibilities in feed composition, validation was carried-out in four different types of feed chosen as representative matrixes. Feed samples intended for use in poultry, pigs, cows, and sheep were selected during the first half of 2019. These samples were kindly donated by different agricultural cooperatives and factories dedicated to feed production in Spain. We collected 100 g samples from each type of feed. The samples were stored at 4 °C until analysis. Table 6 shows the nutritional composition of the analyzed feeds.

### 5.5. Standard Solutions

Two stock solutions containing all the mycotoxins were prepared by diluting different volumes of each mycotoxin solution in ACN. Mycotoxin concentrations in these stock solutions were 12.6 μg/L for AFB2 and AFG2, 40 μg/L for AFB1 and AFG1, 100 μg/L for OTA and OTB, and 840 μg/L for ZEA. To ensure adequate preparation, each stock solution was analyzed by LC-FLD and peak areas for each mycotoxin were compared (Table S7) and an RSD (%) < 1% were found for each one of them. Each stock solution was aliquoted in 1 mL vials and stored at −20 °C. Before use, the corresponding aliquot was kept at room temperature and in darkness for 30 min. Calibrators were prepared daily by evaporating a given volume of the stock solution of mycotoxins at 65 °C under vacuum in an evaporator (Genevac Mivac Duo, Ipswich, UK). The residue was then dissolved in 5 mL of a mixture consisting of 70% water (acidified with 0.1% orthophosphoric acid), 21% MeOH, and 9% ACN.

### 5.6. Sample Preparation

One hundred grams of feed were homogenized prior to the extraction procedure. Homogenization is very important to obtain a representative sample and, additionally, it is also a challenge. In order to achieve it, 100 g of the selected feed samples was crushed in an analytical mill (A 11 basic from IKA^®^-Werke GmbH & Co. KG, Staufen, Germany). The resultant flour like compound was milled once again. From this homogenate, 0.5 g was put into a conical tube (50 mL) and then 5 mL of a solution of ACN/water/orthophosphoric acid (79/20/1) was added. After vortexing (Fisherbrand™ Digital Multi-Tube Vortexer, Fisher Scientific, Bishop Meadow, UK) for 1 h, the mixture was centrifuged at 5500 rpm for 10 min, and 2 mL of the supernatant were then passed through an Oasis PRIME HLB SPE cartridge (Waters, Milford, MA, USA). Subsequently, 0.5 mL of the eluate were put into a conical tube (15 mL) and chloroform (0.5 mL) was added. The mixture was vortexed for 10 s. LC-grade water (5 mL) was then quickly added and the mixture was vortexed once again for 30 s. The resulting emulsion was centrifuged at 7000 rpm for 5 min in order to precipitate the organic solvent. The supernatant was carefully eliminated and 200 μL of the organic phase were placed in a 1.5 mL Eppendorf tube and evaporated at 65 °C in a Genevac Mivac Duo concentrator. Finally, before chromatographic analysis, the residue was redissolved in 200 μL of mobile phase in the initial chromatographic conditions. This solution was filtered (0.45 μm) and subjected to chromatography.

### 5.7. Equipment and Chromatographic Conditions

Chromatographic analysis was carried out in an LC infinity II (Agilent Technologies, Santa Clara, CA, USA) coupled to a FLD. The column was a C18 Cortecs T3 (Waters, Milford, MA, USA), 150 mm × 4.6 mm × 2.7 μm, linked to a C18 Cortecs T3 precolumn (Waters, Milford, MA, USA) measuring 5 mm × 3.9 mm × 2.7 μm. Photoderivatization of AFB1 and AFG1 was performed between the column and the detector using a photochemical reactor with UV-Light UVE (LcTech, Dorfen, Germany) at 254 nm. Chromatographic separation was performed using acidified water (0.1% orthophosphoric acid), ACN, and MeOH in gradient conditions, as shown in Table 7. The injection volume was 40 μL and the flow of the mobile phase was 1.4 mL/min. The column temperature was set at 40 °C and 6 min was required for column re-equilibration (post-time). For the first 26 min, the FLD conditions were λexcitation 365 nm and λemission 440 nm in order to detect AFs, whereas from 26 min to 44 min, λexcitation was 234 nm and λemission was 469 nm to detect OTA, OTB and ZEA.

### 5.8. Validation of the Analytical Method

The methodology was validated following the following EU guidelines: the Commission Regulation No. 401/2006, which establishes the methods for sampling and analysis for the official control of levels of mycotoxins in foodstuffs [50]; the SANTE document [51], which supplements this regulation; and the Commission Decision 2002/657/EC concerning the performance of analytical methods and the interpretation of results [52]. The studied validation parameters were: linearity, selectivity, precision and trueness (within and between-day), recovery (within and between-day), and limits of detection and quantification. Because of the very different compositions of the feed prepared for different species, selectivity, recovery, and limits of detection and quantification were evaluated in feed for poultry, pigs, sheep, and cows.

Selectivity was studied by analyzing calibrators and feed samples before and after being spiked with mycotoxins at different concentrations. Selectivity was ensured if no interferents appeared at the mycotoxin retention times in the non-spiked samples and if the retention times of mycotoxins in the fortified samples corresponded to those in the calibrators, with a tolerance of 5% [52]. In the case of naturally occurring mycotoxin, samples were spiked and only one peak had to be obtained at the retention time of the mycotoxin [52].

Linearity was evaluated preparing seven calibrators in the range of limit of quantification (LOQ) to 10× LOQ for each mycotoxin. The following concentration ranges were prepared: 0.126–1.26 μg/L (equivalent to 1.26–12.6 μg/kg in feed) for AFB2 and AFG2; 0.4–4 μg/L (equivalent to 4–40 μg/kg in feed) for AFB1 and AFG1; 1–10 μg/L (equivalent to 10–100 μg/kg in feed) for OTA and OTB; and, finally, 8.4–84 μg/L (equivalent to 84–840 μg/kg in feed) of ZEA. The calibration curves were repeated on three different days. Linearity was evaluated using the following criteria: relative error of the mean (RE) of less than 15% of the back-calculated concentrations of the calibrators; correlation coefficient greater than 0.990; and slope of the linear calibration curve statistically different from 0 (*p* = 95%). In order to obtain trueness and precision during the linearity experiments, three calibrators were prepared at three levels (LOQ, 2.5× LOQ, and 10× LOQ) in the ranges of each mycotoxin and analyzed in one day (within-day conditions) and on three consecutive days (between-day conditions). Trueness was calculated as the %RE, and repeatability and intermediate precision were calculated as the RSD in %. For both parameters (%RSD and %RE), a value of less than 15% was defined as the criterion for trueness and precision. RE has been calculated as:

(1) $$RE=\frac{\left(nominal\;value-obtained\;value\right)}{obtained\;value}\times 100$$

Furthermore, to study instrumental precision, one calibrator corresponding to the medium level of the different ranges was analyzed 10 consecutive times. The RSD in % of retention times and peak areas had to be less than 2% for all the mycotoxins.

To determine the limit of detection (LOD), several feed samples were fortified at low levels and analyzed. A signal/noise ratio of 3 was chosen as the criterion for selecting the LOD values. In the determination of LOQ, precision and trueness had to be less than 20% and the recovery value had to meet the values established below. The LOQ level was included as the lowest level in all calibration curves.

Recovery and precision of the method was assessed using fortified feed samples (from each of the species studied). Several 0.5 g aliquots of each type of feed were fortified to obtain concentration levels of 1, 2.5, or 5× LOQ for each of the mycotoxins. The volumes used from the stock solution for spiking were 50, 125, and 250 μL, respectively. Each concentration was prepared in triplicate and this procedure was repeated over three days. After fortification, samples were stored overnight at ambient conditions and in darkness in order to promote complete evaporation of the solvent. After this treatment, samples were processed as described above. Recovery was calculated as the ratio between the concentration obtained and the nominal concentration as %. Recovery had to be between 70% and 110% for AFs and ochratoxins, and between 70% and 120% for ZEA [50], with an RSD% below 15%.

## Figures and Tables

**Table 1:** Linearity results for one of the calibration curves obtained for each mycotoxin.

Mycotoxin	Range (ng/mL)	Curve Equation	R^2^	(Slope CI *p* = 95%)	LOD (µg/kg)	RE (%) of B.C.
AFB1	0.4–4	y = 23.71x + 0.91	0.9993	22.99–24.43	2	<5
AFB2	0.126–1.26	y = 58.69x + 0.42	0.9992	56.81–60.56	0.63	<6
AFG1	0.4–4	y = 7.80x + 0.09	0.9993	7.56–9.04	2	<5
AFG2	0.126–1.26	y = 18.88x + 0.37	0.9920	18.28–19.49	0.63	<6
OTA	1–10	y = 7.32x + 1.20	0.9994	7.11–7.54	5	<4
OTB	1–10	y = 6.23x + 0.98	0.9976	5.88–6.58	5	<7
ZEA	8.4–84	y = 0.25x + 1.65	0.9971	0.24–0.27	42	<14

LOD, limit of detection; CI, coefficient interval; B.C., back-calculated concentration; RE, relative error; AFB1, aflatoxin B1; AFB2, aflatoxin B2; AFG1, aflatoxin G1; AFG2, aflatoxin G2; OTA, ochratoxin A; OTB, ochratoxin B; ZEA, zearalenone.

**Table 2:** Precision and trueness.

Mycotoxin	Precision (%RSD)						Trueness (%RE)					
	Within-Day (*n* = 3)			Between-Day (*n* = 9)			Within-Day (*n* = 3)			Between-Day (*n* = 9)		
	LOQ	2.5× LOQ	10× LOQ	LOQ	2.5× LOQ	10× LOQ	LOQ	2.5× LOQ	10× OQ	LOQ	2.5× LOQ	10× LOQ
AFB1	2.1	2.8	2.8	2.6	2.1	1.8	3.6	1.5	0.7	2.1	2.7	0.7
AFB2	3.1	2.1	3.1	2.6	1.9	2,0	4.2	0.2	0.9	1.6	1.0	0.7
AFG1	2.3	2.9	2.4	4.1	3.8	1.5	4.3	0.4	0.6	3.1	0.8	0.5
AFG2	0.9	0.3	0.2	1.7	1.8	0.6	1.7	0.3	0.6	2.4	0.1	0.7
OTA	0.4	2.3	3.7	4.5	2.3	2.4	3.6	1.7	0.8	4.6	1.7	1.1
OTB	2.6	0.3	0.4	2.2	1.9	0.8	5.7	1.8	1.4	6.2	1.5	1.3
ZEA	4.3	3.8	4.9	7.3	4.2	3.0	13.4	0.7	1.5	5.7	0.5	0.8

LOQ, limit of quantification; RSD, relative standard deviation; RE, relative error.

**Table 3:** Recovery data (%) in within-day conditions.

Mycotoxin	Recovery (*n* = 3; %RSD)											
	Poultry			Pigs			Cows			Sheep		
	LOQ	2.5× LOQ	5× LOQ	LOQ	2.5× LOQ	5× LOQ	LOQ	2.5× LOQ	5× LOQ	LOQ	2.5× LOQ	5× LOQ
AFB1	78.5 (4.4)	80.8 (0.9)	77.9 (1.7)	82.3 (2.0)	84.2 (1.7)	86.6 (0.8)	82.5 (1.5)	81.3 (0.6)	82.3 (1.1)	83.5 (0.9)	86.4 (1.3)	80.7 (0.3)
AFB2	87,0 (2.8)	88.4 (0.4)	79.2 (0.1)	88.3 (2.1)	89.4 (1.0)	84.7 (1.1)	85.5 (0.5)	89.6 (1.0)	78.9 (0.7)	88.3 (0.3)	89.1 (0.6)	79.7 (1.4)
AFG1	73.0 (5.1)	72.7 (3.5)	75.5 (2.0)	80.7 (1.2)	75.8 (0.2)	76.2 (0.7)	70.0 (1.3)	75.5 (1.3)	72.4 (0.5)	76.7(0.9)	79.3 (1.5)	74.6 (0.3)
AFG2	86.1 (3.3)	81.6 (2,0)	81.6 (1.0)	74.2 (2.6)	79.3 (3.2)	84.7 (0.3)	82.6 (1.1)	84.6 (1.5)	78.9 (0.2)	89.5 (0.5)	81.3 (1.2)	78.6 (0.3)
OTA	83.4 (3.1)	80.4 (1.1)	79.5 (1.0)	87.5 (2.3)	84.6 (1.6)	87.1 (1.5)	83.8 (1.9)	86.5 (2.0)	81.7 (1.7)	85.8 (2.6)	82.4 (5.0)	81.1 (0.4)
OTB	82.4 (3.9)	85.1 (0.2)	74.9 (0.8)	85.1 (3.4)	75.6 (0.9)	77.6 (1.3)	79.5 (3.3)	76.4 (4.7)	75.0 (2.1)	76.6 (2.1)	74.2 (1.6)	73.4 (0.7)
ZEA	79.0 (0.8)	74.3 (3.0)	76.6 (0.6)	73.3 (1.3)	72.9 (4.2)	77.0 (1.0)	71.2 (3.8)	73.7 (1.6)	77.8 (0.7)	75.2 (4.6)	73.0 (1.6)	80.2 (0.5)

LOQ, limit of quantification; RSD, relative standard deviation.

**Table 4:** Recovery data (%) in between-day conditions.

Mycotoxin	Recovery Between-Day (*n* = 9; %RSD)											
	Poultry			Pigs			Cows			Sheep		
	LOQ	2.5× LOQ	5× LOQ	LOQ	2.5× LOQ	5× LOQ	LOQ	2.5× LOQ	5× LOQ	LOQ	2.5× LOQ	5× LOQ
AFB1	81.1 (3.5)	81.7 (1.4)	77.1 (1.6)	82.9 (1.3)	81.9 (2.4)	84.8 (1.8)	82.6 (1.2)	83.8 (2.4)	83.1 (1.2)	82.3 (2.0)	85.6 (1.2)	81.4 (1.2)
AFB2	87.0 (2.5)	88.4 (1.0)	79.2 (2.1)	88.8 (1.9)	91.8 (2.4)	83.4 (1.7)	86.4 (1.6)	92.7 (2.6)	79.2 (1.0)	86.9 (1.8)	90.7 (3.3)	80.1 (0.9)
AFG1	73.0 (3.1)	72.7 (4.2)	75.5 (2.3)	77.3 (6.2)	74.9 (2.3)	75.7 (1.1)	72.6 (3.8)	74.6 (2.8)	73.6 (2.8)	75.7 (2.0)	78.0 (2.0)	75.6 (2.8)
AFG2	86.1 (4.4)	81.6 (2.2)	81.6 (1.7)	78.5 (4.6)	79.1 (2.8)	83.3 (1.8)	83.6 (4.9)	83.7 (1.9)	78.5 (1.4)	86.4 (5.2)	80.7 (2.2)	78.9 (1.2)
OTA	83.4 (2.3)	80.4 (2.0)	79.5 (2.1)	86.0 (2.4)	85.5 (1.8)	85.8 (2.8)	82.9 (2.2)	86.6 (1.5)	81.2 (1.0)	85.7 (2.0)	82.9 (2.7)	80.5 (1.7)
OTB	82.4 (4.4)	85.1 (3.0)	74.9 (1.8)	84.2 (3.2)	76.8 (1.6)	78.4 (1.4)	79.6 (3.2)	77.3 (2.6)	76.2 (1.6)	77.0 (2.7)	75.5 (1.8)	76.1 (2.9)
ZEA	79.0 (3.6)	74.3 (1.7)	76.6 (0.8)	75.6 (3.0)	73.5 (2.3)	77.8 (1.8)	73.6 (3.8)	73.7 (1.8)	77.8 (0.8)	75.2 (3.0)	72.9 (2.1)	78.9 (1.7)

LOQ, limit of quantification; RSD, relative standard deviation.

**Table 5:** Global Recovery data (%).

Mycotoxin	Poultry (*n* = 27; RSD%)	Pigs (*n* = 27; RSD%)	Cows (*n* = 27; RSD%)	Sheep (*n* = 27; RSD%)
AFB1	80.0 (3.4)	83.2 (2.3)	83.2 (1.7)	83.1 (2.7)
AFB2	84.9 (5.2)	88.0 (4.4)	86.1 (6.8)	85.9 (5.7)
AFG1	73.6 (3.6)	75.9 (4.0)	73.6 (3.2)	76.4 (2.7)
AFG2	83.1 (3.9)	80.3 (4.1)	81.9 (4.3)	82.0 (5.2)
OTA	82.6 (3.8)	85.8 (2.3)	83.6 (3.2)	83.0 (3.4)
OTB	79.2 (5.0)	79.8 (4.6)	77.7 (3.1)	76.2 (2.5)
ZEA	76.7 (3.4)	75.6 (3.3)	75.1 (3.5)	75.7 (4.0)

**Table 6:** Nutritional composition of the analyzed feeds.

Type of Feed	Protein%	Fat%	Ash%	Crude Fiber%
Cows	11.14	4.14	5.04	7.22
Sheep	10.89	3.01	6.22	12.20
Pigs	20.21	4.61	9.66	8.82
Poultry	15.34	5.09	4.65	3.89

**Table 7:** Gradient for the chromatographic separation.

Time (min)	%Acidified Water	%ACN	%MeOH
0.00	70.0	9.0	21.0
12.55	73.0	9.0	18.0
21.70	73.0	9.0	18.0
21.75	65.0	9.0	26.0
22.50	65.0	9.0	26.0
26.50	45.0	16.5	38.5
36.80	45.0	16.5	38.5
36.85	10.0	45.0	45.0
44.00	10.0	45.0	45.0

ACN, acetonitrile; MeOH, methanol.

## Supplementary Materials

The following are available online at https://www.mdpi.com/2072-6651/12/6/374/s1, Table S1: Retention time (min) obtained in selectivity studies; Table S2: Calibration curves; Table S3: Raw data for recovery (%) in poultry feed; Table S4: Raw data for recovery (%) in pig feed; Table S5: Raw data for recovery (%) in cow feed; Table S6: Raw data for recovery (%) in sheep feed; Table S7: Raw data (areas) obtained in the two prepared mixed stock solutions.

## Abbreviations

ACN	acetonitrile
AFB1	aflatoxin B1
AFB2	aflatoxin B2
AFG1	aflatoxin G1
AFG2	aflatoxin G2
AFs	aflatoxins
DLLME	dispersive liquid–liquid microextraction
EU	European Union
FB1	fumonisin B1
FB2	fumonisin B2
FLD	fluorescence detector
IAC	immunoaffinity column
LC	liquid chromatography
LC-MS/MS	LC coupled with MS in tandem
LOD	limit of detection
LOQ	limit of quantification
MeOH	methanol
MS	mass spectrometry detector
OTA	ochratoxin A
OTB	ochratoxin B
PBS	phosphate buffer
QuEChERS	Quick, Easy, Cheap, Effective, Rugged and Safe
rpm	revolutions per minute
RE	relative error
RSD	relative standard deviation
SPE	solid-phase extraction
TFA	trifluoroacetic acid
ZEA	zearalenone
