# Supplementary material for: Mycotoxin Determination in Animal Feed: An LC-FLD Method for Simultaneous Quantification of Aflatoxins, Ochratoxins and Zearelanone in This Matrix
Source: Toxins (Basel). 2020 Jun 5;12(6):374. doi: 10.3390/toxins12060374 (PMC7354491; doi:10.3390/toxins12060374)
Supplement: Supplementary file 1 [file toxins-12-00374-s001.pdf]

Supplementary Materials: Mycotoxin Determination in Animal Feed: An LC-FLD Method for Simultaneous Quantification of Aflatoxins, Ochratoxins and Zearelanone in This Matrix

Borja Muñoz-Solano and Elena González-Peñas

Table S1. Retention time (min) obtained in selectivity studies.

Mycotoxin		Calibrators	Poultry Feed	Pigs Feed	Cows Feed	Sheep Feed
AFG2	Mean t_R (n = 9)	10.487	10.561	10.575	10.555	10.551
	RSD (%)	0.6	0.5	0.5	0.4	0.4
	RE (%)		0.7	0.9	0.7	0.6
AFG1	Mean t_R (n = 9)	14.376	14.463	14.478	14.500	14.456
	RSD (%)	0.2	0.4	0.5	0.3	0.3
	RE (%)		0.6	0.7	0.9	0.6
AFB2	Mean t_R (n = 9)	18.280	18.390	18.300	18.366	18.311
	RSD (%)	0.3	0.2	0.2	0.3	0.2
	RE (%)		0.6	0.1	0.5	0.2
AFB1	Mean t_R (n = 9)	24.795	24.815	24.825	24.831	24.834
	RSD (%)	0.2	0.2	0.2	0.2	0.2
	RE (%)		0.1	0.1	0.2	0.2
OTB	Mean t_R (n = 9)	31.327	31.364	31.364	31.363	31.352
	RSD (%)	0.3	0.2	0.1	0.2	0.2
	RE (%)		0.1	0.1	0.1	0.1
ZEA	Mean t_R (n = 9)	34.957	34.984	34.990	34.973	34.974
	RSD (%)	0.2	0.1	0.2	0.2	0.2
	RE (%)		0.1	0.1	0.1	0.1
OTA	Mean t_R (n = 9)	37.597	37.626	37.591	37.573	37.583
	RSD (%)	0.1	0.1	0.1	0.1	0.1
	RE (%)		0.1	0.1	0.1	0.1

t_R (n = 9) n = 9 retention time. RSD, relative standard deviation; RE, relative error; AFB1, aflatoxin B1; AFB2, aflatoxin B2; AFG1, aflatoxin G1; AFG2, aflatoxin G2; OTA, ochratoxin A; OTB, ochratoxin B; ZEA, zearalenone.

Table S2. Calibration curves.

Mycotoxin	Calibration Range (ng/mL)	Day 1	Day 2	Day 3
AFG2	0.126–1.26	y = 18.88x + 0.37<br>Slope CI = 18.28–19.49<br>R^2 = 0.9920	y = 18.91x + 0.33<br>Slope CI = 18.43–19.40<br>R^2= 0.9995	y = 19.95x + 0.20<br>Slope CI= 19.29–20.61<br>R^2 = 0.9992
AFG1	0.40–4.00	y = 7.80x + 0.09<br>Slope CI = 7.56–9.04<br>R^2= 0.9993	y = 7.68x + 0.10<br>Slope CI = 7.50–7.87<br>R^2 = 0.9995	y = 8.13x − 0.20<br>Slope CI= 7.85–8.41<br>R^2 = 0.9991
AFB2	0.126–1.26	y = 58.69x + 0.42<br>Slope CI= 56.81–60.56<br>R^2 = 0.9992	y = 55.07x + 0.26<br>Slope CI= 54.40–55.74<br>R^2= 0.9999	y = 58.11x − 0.16<br>Slope CI= 56.08–60.14<br>R^2= 0.9991
AFB1	0.40–4.00	y = 23.71x + 0.91<br>Slope CI= 22.99–24.43<br>R^2 = 0.9993	y = 22.74x + 0.27<br>Slope CI = 22.13–23.36<br>R^2= 0.9994	y = 23.56x − 0.39<br>Slope CI= 23.28–23.94<br>R^2= 0.9994
OTB	1.0–10.0	y = 6.23x + 0.98<br>Slope CI= 5.88–6.58<br>R^2= 0.9976	y = 6.23x + 0.93<br>Slope CI= 5.96–6.51<br>R^2= 0.9985	y = 6.22x + 0.92<br>Slope CI= 5.89–6.56<br>R^2= 0.9978
ZEA	8.4–84	y = 0.25x + 1.65<br>Slope CI= 0.24–0.27<br>R^2= 0.9971	y = 0.26x + 1.30<br>Slope CI= 0.25–0.27<br>R^2 = 0.9982	y = 0.24x + 1.31<br>Slope CI= 0.22–0.25<br>R^2 = 0.9963
OTA	1.0–10.0	y = 7.32x + 1.20<br>Slope CI = 7.11–7.54<br>R^2= 0.9994	y = 6.62x + 1.27<br>Slope CI = 6.41–6.83<br>R^2 = 0.9992	y = 6.73x + 1.39<br>Slope CI= 6.26–7.20<br>R^2 = 0.9963

CI, confidence interval (p = 95%).

Table S3. Raw data for recovery (%) in poultry feed.

	AFB1	AFB2	AFG1	AFG2	OTA	OTB	ZEA
**LOQ**							
Day 1	81.5	86.5	74.0	91.2	81.9	75.1	76.9
	79.3	88.9	76.1	88.3	84.5	78.4	76.3
	74.8	91.3	68.8	85.5	87.1	81.6	75.7
Day 2	82.0	86.6	72.2	86.9	83.0	85.5	84.4
	85.1	87.2	74.3	79.9	84.1	84.6	80.6
	81.7	86.1	71.8	82.7	83.8	84.0	79.5
Day 3	82.3	85.2	73.1	83.2	81.8	84.0	81.4
	81.0	86.6	75.4	90.9	83.3	86.6	77.1
	82.0	84.1	71.8	86.3	80.4	81.8	79.2
Within-day recovery (n = 3, first three dataset)							
Mean	78.5	87,0	73.0	86.1	83.4	82.4	79.0
RSD (%)	4.4	2.8	5.1	3.3	3.1	3.9	0.8
Between-day recovery (n = 9)							
Mean	81.1	87.0	73.0	86.1	83.4	82.4	79.0
RSD (%)	3.5	2.4	3.0	4.4	2.3	4.4	3.6
**2.5× LOQ**							
Day 1	81.5	88.2	74.1	84.1	78.2	82.6	72.4
	80.8	88.5	71.6	81.0	79.2	82.8	74.7
	80.1	88.9	69.1	83.4	80.1	83.0	76.9
Day 2	81.8	86.9	69.3	81.7	79.2	82.5	74.7
	81.1	88.6	70.4	80.1	80.1	84.1	74.0
	81.8	88.0	70.9	82.5	79.8	87.8	73.1
Day 3	81.9	87.6	75.7	79.7	83.5	88.5	73.8
	82.5	88.7	76.2	79.0	81.6	87.0	75.3
	84.1	89.9	76.8	82.9	81.8	87.3	74.3
Within-day recovery (n = 3, first three dataset)							
Mean	80.8	88.4	72.7	81.6	80.4	85.1	74.3
RSD (%)	0.9	0.4	3.5	2.0	1.1	0.2	3.0
Between-day recovery (n = 9)							
Mean	81.7	88.4	72.7	81.6	80.4	85.1	74.3
RSD (%)	1.4	1.0	4.2	2.2	2.0	3.0	1.7
**5× LOQ**							
Day 1	79.4	78.8	73.9	82.1	80.5	77.2	75.5
	77.5	78.9	72.1	82.9	79.7	76.2	75.9
	76.9	79.0	75.1	83.7	79.0	76.1	76.3
Day 2	77.8	82.4	75.8	80.8	80.6	73.9	77.2
	78.1	80.6	77.7	81.1	79.5	73.4	76.5
	77.1	80.3	77.0	82.6	79.7	74.0	77.3
Day 3	75.8	78.0	77.1	79.8	80.4	74.3	77.3
	75.9	78.2	75.3	79.9	75.4	74.9	76.9
	75.7	76.9	75.3	81.4	80.3	73.9	76.6
Within-day recovery (n = 3, first three dataset)							
Mean	77.9	79.2	75.5	81.6	79.5	74.9	76.6
RSD (%)	1.7	0.1	2.0	1.0	1.0	0.8	0.6
Between-day recovery (n = 9)							
Mean	77.1	79.2	75.5	81.6	79.5	74.9	76.6
RSD (%)	1.6	2.1	2.3	1.7	2.1	1.8	0.8

RSD, relative standard deviation; LOQ, limit of quantification.

Table S4. Raw data for recovery (%) in pig feed.

	**AFB1**	**AFB2**	**AFG1**	**AFG2**	**OTA**	**OTB**	**ZEA**
**LOQ**							
Day 1	83.9	86.6	79.8	72.3	85.2	88.2	74.3
	82.1	90.3	80.6	74.2	89.1	84.6	72.4
	80.8	88.0	81.6	76.2	88.1	82.6	73.3
Day 2	83.0	89.4	78.6	78.4	84.3	84.0	79.3
	83.9	88.2	81.6	78.7	84.0	81.9	76.9
	83.0	90.5	78.6	82.6	83.3	80.7	74.0
Day 3	82.4	91.1	75.4	81.0	86.7	85.3	75.7
	84.1	88.2	67.5	80.5	85.9	82.5	77.3
	82.6	86.8	72.1	82.3	87.8	88.2	76.9
Within-day recovery (*n* = 3, first three dataset)							
Mean	82.3	88.3	80.7	74.2	87.5	85.1	73.3
RSD (%)	2.0	2.1	1.2	2.6	2.3	3.4	1.3
Between-day recovery (*n* = 9)							
Mean	82.9	88.8	77.3	78.5	86.0	84.2	75.6
RSD (%)	1.3	1.9	6.2	4.6	2.4	3.2	3.0
**2.5× LOQ**							
Day 1	83.8	90.4	75.6	82.1	83.5	75.3	69.4
	85.8	89.0	75.8	78.5	86.1	75.2	74.8
	83.1	88.7	75.9	77.2	84.1	76.4	74.5
Day 2	80.5	94.8	75.8	81.0	85.7	75.9	73.7
	81.7	94.0	76.9	77.8	85.5	76.7	73.3
	79.8	92.5	75.4	76.9	84.8	77.6	72.6
Day 3	80.9	93.6	72.9	77.1	87.8	77.7	74.2
	79.9	91.2	73.5	78.4	87.8	77.6	75.1
	81.7	91.7	71.8	82.6	84.4	78.9	73.8
Within-day recovery (*n* = 3, first three dataset)							
Mean	84.2	89.4	75.8	79.3	84.6	75.6	72.9
RSD (%)	1.7	1.0	0.2	3.2	1.6	0.9	4.2
Between-day recovery (*n* = 9)							
Mean	81.9	91.8	74.9	79.1	85.5	76.8	73.5
RSD (%)	2.4	2.4	2.3	2.8	1.8	1.6	2.3
**5× LOQ**							
Day 1	86.1	85.6	76.6	84.5	88.5	76.9	77.8
	87.4	84.7	75.6	85.0	86.7	78.7	76.5
	86.4	83.7	76.4	84.7	86.1	77.2	76.6
Day 2	84.3	83.7	76.5	84.0	83.9	78.6	76.8
	83.8	83.4	75.9	83.2	85.2	77.2	78.0
	84.8	84.1	75.1	83.7	90.5	79.8	81.0
Day 3	83.6	83.1	74.4	81.6	84.3	78.7	77.4
	84.1	81.5	76.0	82.3	83.5	78.9	78.1
	82.6	81.0	74.4	80.7	83.4	79.9	78.2
Within-day recovery (*n* = 3, first three dataset)							
Mean	86.6	84.7	76.2	84.7	87.1	77.6	77.0
RSD (%)	0.8	1.1	0.7	0.3	1.5	1.3	1.0
Between-day recovery (*n* = 9)							
Mean	84.8	83.4	75.7	83.3	85.8	78.4	77.8
RSD (%)	1.8	1.7	1.1	1.8	2.8	1.4	1.8

RSD, relative standard deviation.

Table S5. Raw data for recovery (%) in cow feed.

	AFB1	AFB2	AFG1	AFG2	OTA	OTB	ZEA
**LOQ**							
	82.3	85.2	70.8	82.5	81.9	82.5	74.0
Day 1	83.7	85.4	69.0	83.5	84.7	78.1	71.0
	81.4	86.0	70.3	81.7	84.7	77.9	68.6
	84.4	84.6	69.7	79.6	85.3	83.8	76.9
Day 2	82.8	88.4	75.4	82.5	84.0	81.1	74.1
	81.5	86.9	72.2	78.4	82.2	80.9	71.7
	82.2	88.6	74.2	84.6	81.5	78.4	77.1
Day 3	82.0	86.6	76.1	92.0	81.3	77.1	74.1
	83.2	85.6	75.4	87.2	80.4	76.5	75.2
Within-day recovery (*n* = 3, first three dataset)							
Mean	82.5	85.5	70.0	82.6	83.8	79.5	71.2
RSD (%)	1.5	0.5	1.3	1.1	1.9	3.3	3.8
Between-day recovery (*n* = 9)							
Mean	82.6	86.4	72.6	83.6	82.9	79.6	73.6
RSD (%)	1.2	1.6	3.8	4.9	2.2	3.2	3.8
**2.5× LOQ**							
	81.4	89.2	74.4	83.3	84.5	72.4	74.9
Day 1	80.8	90.7	76.3	85.8	87.9	79.3	73.8
	81.7	88.9	75.8	84.8	87.0	77.6	72.5
	85.2	94.2	71.1	83.5	87.2	77.7	73.7
Day 2	84.6	93.6	74.2	85.2	84.7	78.3	74.0
	84.2	94.3	75.3	80.9	87.8	77.6	71.2
	86.0	94.2	71.8	82.3	86.7	77.0	75.1
Day 3	85.6	94.5	77.6	83.3	87.8	79.1	73.1
	85.0	94.8	75.3	84.7	86.3	76.9	75.3
Within-day recovery (*n* = 3, first three dataset)							
Mean	81.3	89.6	75.5	84.6	86.5	76.4	73.7
RSD (%)	0.6	1.0	1.3	1.5	2.0	4.7	1.6
Between-day recovery (*n* = 9)							
Mean	83.8	92.7	74.6	83.7	86.6	77.3	73.7
RSD (%)	2.4	2.6	2.8	1.9	1.5	2.6	1.8
**5× LOQ**							
	83.3	78.4	72.8	78.7	83.2	75.2	77.9
Day 1	81.7	79.5	72.1	79.1	80.4	76.4	78.2
	81.9	78.8	72.4	79.0	81.3	73.3	77.2
	83.6	80.4	74.4	79.9	81.1	77.0	77.6
Day 2	83.6	80.1	75.1	78.4	81.6	76.9	78.0
	81.7	79.6	69.8	76.0	80.7	76.3	77.1
	84.3	78.7	73.9	78.2	81.1	76.9	77.3
Day 3	84.1	79.3	75.1	78.5	80.5	76.6	78.2
	83.6	78.2	76.5	78.2	80.8	76.9	79.0
Within-day recovery (*n* = 3, first three dataset)							
Mean	82.3	78.9	72.4	78.9	81.7	75.0	77.8
RSD (%)	1.1	0.7	0.5	0.2	1.7	2.1	0.7
Between-day recovery (*n* = 9)							
Mean	83.1	79.2	73.6	78.5	81.2	76.2	77.8
RSD (%)	1.2	1.0	2.8	1.4	1.0	1.6	0.8

RSD, relative standard deviation.

Table S6. Raw data for recovery (%) in sheep feed.

	**AFB1**	**AFB2**	**AFG1**	**AFG2**	**OTA**	**OTB**	**ZEA**
**LOQ**							
Day 1	83.5	87.9	76.1	89.6	86.6	76.6	71.5
	84.3	88.5	77.5	89.9	83.3	78.1	75.7
	82.9	88.3	76.4	89.1	87.5	75.0	78.4
Day 2	80.7	83.8	74.4	84.7	84.0	76.6	74.3
	82.0	86.1	77.2	86.5	85.3	75.5	76.9
	79.3	87.9	75.6	90.5	88.8	74.1	72.5
Day 3	81.5	85.4	76.1	75.9	85.4	78.3	75.9
	84.1	86.5	75.3	84.7	85.1	78.1	77.3
	82.7	88.0	72.6	86.3	85.7	81.0	74.0
Within-day recovery (*n* = 3, first three dataset)							
Mean	83.5	88.3	76.7	89.5	85.8	76.6	75.2
RSD (%)	0.9	0.3	0.9	0.5	2.6	2.0	4.6
Between-day recovery (*n* = 9)							
Mean	82.3	86.9	75.7	86.4	85.7	77.0	75.2
RSD (%)	2.0	1.8	2.0	5.2	2.0	2.7	3.0
**2.5× LOQ**							
Day 1	85.6	89.1	78.4	80.2	83.0	73.5	73.4
	87.7	89.7	80.7	82.0	78.0	75.6	71.7
	86.0	88.6	78.9	81.7	86.1	73.6	73.9
Day 2	85.2	88.8	77.1	79.9	85.4	77.6	72.8
	84.7	88.7	79.2	80.1	83.1	75.7	72.1
	83.8	87.6	78.5	81.7	82.3	76.8	74.6
Day 3	85.6	94.6	77.1	83.1	82.4	75.9	75.1
	85.7	94.8	76.6	80.8	82.9	75.8	72.5
	86.1	94.7	75.4	77.0	82.7	75.1	70.1
Within-day recovery (*n* = 3, first three dataset)							
Mean	86.4	89.1	79.3	81.3	82.4	74.2	73.0
RSD (%)	1.3	0.6	1.5	1.2	5.0	1.6	1.6
Between-day recovery (*n* = 9)							
Mean	85.6	90.7	78.0	80.7	82.9	75.5	72.9
RSD (%)	1.2	3.3	2.0	2.2	2.7	1.8	2.1
**5× LOQ**							
Day 1	80.5	78.6	74.4	78.3	80.8	73.3	79.7
	80.8	80.7	74.6	78.8	81.3	72.9	80.2
	81.0	79.8	74.8	78.6	81.1	73.9	80.6
Day 2	81.8	80.6	76.1	79.3	80.6	76.7	78.9
	82.1	79.7	80.7	80.4	80.4	76.7	79.1
	79.7	80.9	75.3	79.9	76.9	76.6	78.0
Day 3	82.2	80.4	75.1	78.6	81.3	77.5	79.8
	82.8	79.8	76.0	78.5	81.1	78.4	77.3
	81.7	80.7	73.1	77.3	80.7	78.7	76.8
Within-day recovery (*n* = 3, first three dataset)							
Mean	80.7	79.7	74.6	78.6	81.1	73.4	80.2
RSD (%)	0.3	1.4	0.3	0.3	0.4	0.7	0.5
Between-day recovery (*n* = 9)							
Mean	81.4	80.1	75.6	78.9	80.5	76.1	78.9
RSD (%)	1.2	0.9	2.8	1.2	1.7	2.9	1.7

RSD, relative standard deviation.

Table S7. Raw data (areas) obtained in the two prepared mixed stock solutions.

	AFB1	AFB2	AFG1	AFG2	OTA	OTB	ZEA.
100× LOQ	980.4	699.8	320.7	271.9	800.1	677.9	349.9
	972.2	690.9	319.7	275.4	796.3	669.7	352.3
Mean	976.3	695.35	320.2	273.65	798.2	673.8	351.1
RSD (%)	0.6	0.9	0.2	0.9	0.3	0.9	0.5

RSD, relative standard deviation.
